# Homogeneous MGMT Immunoreactivity Correlates with an Unmethylated MGMT Promoter Status in Brain Metastases of Various Solid Tumors

**DOI:** 10.1371/journal.pone.0004775

**Published:** 2009-03-10

**Authors:** Barbara Ingold, Peter Schraml, Frank L. Heppner, Holger Moch

**Affiliations:** 1 Institute of Surgical Pathology, Department of Pathology, University Hospital Zurich, Zurich, Switzerland; 2 Institute of Neuropathology, Department of Pathology, University Hospital Zurich, Zurich, Switzerland; Ordway Research Institute, United States of America

## Abstract

The O^6^-methylguanine-methyltransferase (MGMT) promoter methylation status is a predictive parameter for the response of malignant gliomas to alkylating agents such as temozolomide. First clinical reports on treating brain metastases with temozolomide describe varying effects. This may be due to the fact that MGMT promoter methylation of brain metastases has not yet been explored in depth. Therefore, we assessed MGMT promoter methylation of various brain metastases including those derived from lung (n = 91), breast (n = 72) kidney (n = 49) and from malignant melanomas (n = 113) by methylation-specific polymerase chain reaction (MS-PCR) and MGMT immunoreactivity. Fifty-nine of 199 brain metastases (29.6%) revealed a methylated MGMT promoter. The methylation rate was the highest in brain metastases derived from lung carcinomas (46.5%) followed by those from breast carcinoma (28.8%), malignant melanoma (24.7%) and from renal carcinoma (20%). A significant correlation of homogeneous MGMT-immunoreactivity (>95% MGMT positive tumor cells) and an unmethylated MGMT promoter was found. Promoter methylation was detected in 26 of 61 (43%) tumors lacking MGMT immunoreactivity, in 17 of 63 (27%) metastases with heterogeneous MGMT expression, but only in 5 of 54 brain metastases (9%) showing a homogeneous MGMT immunoreactivity. Our results demonstrate that a significant number of brain metastases reveal a methylated MGMT-promoter. Based on an obvious correlation between homogeneous MGMT immunoreactivity and unmethylated MGMT promoter, we hypothesize that immunohistochemistry for MGMT may be a helpful diagnostic tool to identify those tumors that probably will not benefit from the use of alkylating agents. The discrepancy between promoter methylation and a lack of MGMT immunoreactivity argues for assessing MGMT promoter methylation both by immunohistochemical as well as by molecular approaches for diagnostic purposes.

## Introduction

O^6^-methylguanine-methyltransferase (MGMT) is a DNA repair protein which catalyzes the transfer of the methyl group from O^6^-methylguanine to a cysteine residue of its active site [1]. In this single step reaction, DNA-lesions caused by alkylating substances are repaired. MGMT subsequently is ubiquitylated and degraded [2]. Therefore, the cellular activity of MGMT is directly linked to the expression level of the protein. The high DNA repair activity of tumor cells expressing active MGMT is believed to defend the tumor from the cytotoxic effects of alkylating agents [3], [4]. Tumors with low or no levels of MGMT due to epigenetic silencing of MGMT by methylation of CpG islands in the promoter region may predictably be responsive to such therapy [5]. Chemotherapy-induced lesions remain un-repaired and trigger cytotoxicity and apoptosis, which is the desired outcome. In several studies the correlation of MGMT promoter methylation status and the response of tumors to alkylating agents (e.g. carmustin, lomustine, temozolomide) has been examined [5]–[7]. For example patients suffering from glioblastoma multiforme with a methylated MGMT promoter had a better outcome after therapy with temozolomide (TMZ) than those patients, without a methylated MGMT promoter. This supports the hypothesis that MGMT inactivation by aberrant promoter methylation correlates with the sensitivity of the tumor to alkylating agents [7].

The most common intracranial neoplasms of the adult are metastases originating from primary systemic neoplasms [8]. The most frequent primary sources of brain metastases are carcinomas of the respiratory tract (50%) and breast (15%) followed by malignant melanomas (10.5%) [8]. Brain metastases of renal cancer have been reported in up to 5%. In about 10% the metastatic origin remains unknown. A broad range of incidence and prevalence is reported for all types of brain metastases, since calculations are based on assorted epidemiologic, autoptic and clinical studies [9].

The ability to effectively treat brain metastases, however, remains poor. Surgery is limited due to the delicate structure of the human brain which excludes functionally important areas of resection, and the risk of neurotoxic side effects, especially in elderly patients and children, limits the tolerance of radiation [10]. So far, chemotherapy had played a minor role in the treatment of brain metastases and its profit is yet not fully defined. The blood-brain-barrier has been the major obstacle to successfully deliver active chemotherapeutic agents. Moreover, the limited benefit derived from chemotherapy is associated with severe side effects [11]. TMZ is an orally administered alkylating agent that plays an important role in the standard therapy of malignant gliomas. It has a good blood-brain-barrier penetration which results in therapeutic concentrations within the central nervous system (CNS) and confers manageable side effects. The possible role of TMZ in the treatment of brain metastases is currently being explored. Several studies on utilizing TMZ in patients with brain metastases describe rather variable outcomes [12]. Although MGMT promoter methylation is known to be a predictive factor for the success of using alkylating substances like TMZ in malignant gliomas [4], [5], [7], MGMT promoter methylation of brain metastases has not been explored in depth.

Most studies on MGMT promoter methylation rely on the methylation-specific polymerase chain reaction assay (MS-PCR) [5], [7], [13]. Other investigators prefer the somewhat simpler approach to detect the function of the MGMT gene by means of immunhistochemistry [14]–[16]. However, data addressing both, MGMT promoter methylation and MGMT immunoreactivity, are sparse and controversially discussed [17]–[19]. Consequently, we aimed here to investigate comprehensively MGMT promoter methylation and MGMT immunohistochemistry in brain metastases derived from lung, breast and renal cell carcinomas as well as from malignant melanomas.

## Materials and Methods

### Tumors

Formalin-fixed, paraffin-embedded tissue samples of 325 brain metastases were subjected for MGMT promoter methylation analysis and immunohistochemical analysis comprising brain metastases of carcinomas of the lung (n = 91), the breast (n = 72) and the kidney (n = 49, clear cell renal cell carcinoma) as well as malignant melanoma (n = 113). Brain metastases were derived from the Institute of Neuropathology, University of Zurich (1981–2005). All tumor samples have been re-evaluated systematically by one neuropathologist (FLH). This project has been approved by the local ethics committee (ref. number StV 37-2005).

### DNA extraction and methylation-specific PCR

Genomic DNA was isolated from two to three 20 µm thick paraffin sections by the EZ1 DNA tissue kit (Qiagen) using the BioRobot EZ1 workstation (Qiagen). Sodium bisulfite modification of isolated DNA was performed using the Zymo research EZ DNA Methylation kit (Zymo Research, Orange, CA, USA). The analysis of the methylation status of the MGMT gene was done in a nested, two stage PCR approach basically as reported by Palmisano and colleagues [13]. DNA of normal lymphocytes was used as negative control for methylated alleles of MGMT, and DNA of the cell-line SW620 (human colorectal adenocarcinoma; American Type Culture Collection) was used as positive control for methylated alleles of MGMT. PCR products were analyzed by electrophoresis in 2.5% agarose gel containing ethidium bromide.

### Tissue microarray construction

Formalin-fixed, paraffin-embedded tissue samples were used to generate tissue microarrays (TMA) as described previously [20]–[23]. A morphologically representative region of the paraffin ‘donor’ block was chosen. Tissue cylinders were punched from this area (diameter: 0.6 mm) and precisely arrayed into a new ‘recipient’ paraffin block using a customer built instrument. After completing the block construction, four micrometer sections of the resulting tumor tissue microarray block were cut for further analysis.

### Immunohistochemistry

Immunohistochemistry was performed using the Bond™ automated staining system (Vision BioSystems, Newcastle Upon Tyne, UK). Sections were incubated with an antibody against the human MGMT (dilution: 1∶160; monoclonal mouse IgG1; clone MT3.1; NeoMarkers, Newmarket, UK). For antigen retrieval, slides were pre-treated with the Bond™ Epitope Retrieval Solution 2 (Vision BioSystems). Endogenous biotin was blocked with the appropriate kit. Slides were incubated with the Bond™ Polymer Refine Detection kit (Vision BioSystems). Slides were counterstained with hematoxylin prior to glass coverslipping. MGMT-immunopositive cells revealed a strong nuclear staining. Lymphocytes and endothelial cells served as internal positive control. The immunoreactivity was scored semi-quantitatively as follows: 0: <5% positive tumor cells, 1+: 5–75% positive tumor cells, 2+: 75–95% positive tumor cells, 3+: >95% positive tumor cells. 3+ scores were designated as a homogeneous MGMT expression. Only a nuclear staining was regarded as positive. Colon carcinoma tissue served as positive control.

### Statistical analyses

Contingency table analysis and Chi-square tests were applied for evaluating correlations between MGMT immunoreactivity and promoter methylation using SPSS 15.0 for Windows (SPSS Inc., Chicago, IL). p-values less than 0.05 were considered as significant.

## Results

### MGMT promoter methylation

**Figure 1:**
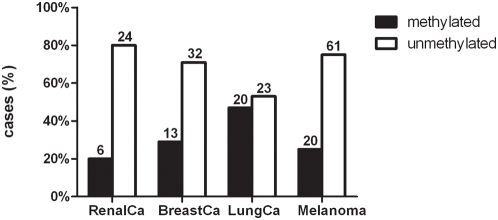
Frequencies of unmethylated and methylated MGMT promoter in the 4 tumor subgroups.

An appropriate amount of DNA with sufficient quality needed for bisulfite conversion could be isolated from 246 of 325 brain metastases (75.7%). DNA was available from brain metastases of lung (n = 63), breast (n = 51), renal cancer (n = 34) and malignant melanoma (n = 98). MGMT methylation status could be determined for 199 of 246 (80.9%) samples. Overall, a methylated MGMT promoter was detectable in 59 of 199 (29.6%) of the metastases by MS-PCR. The frequencies of methylated and unmethylated MGMT promoter in the 4 tumor subgroups is shown in **figure 1**. No subtype specific MGMT promoter methylation differences were detected in brain metastases deriving from lung (squamous cell carcinoma (3), small cell carcinoma (4), adenocarcinoma (16), large cell carcinoma (1), neuroendocrine tumours (2), poorly differentiated (8), NOS (9)) and breast carcinoma (invasive ductal (9), neuroendocrine tumors (2), mucinous carcinoma (1), poorly differentiated (6), NOS (27)).

### MGMT protein expression

**Figure 2:**
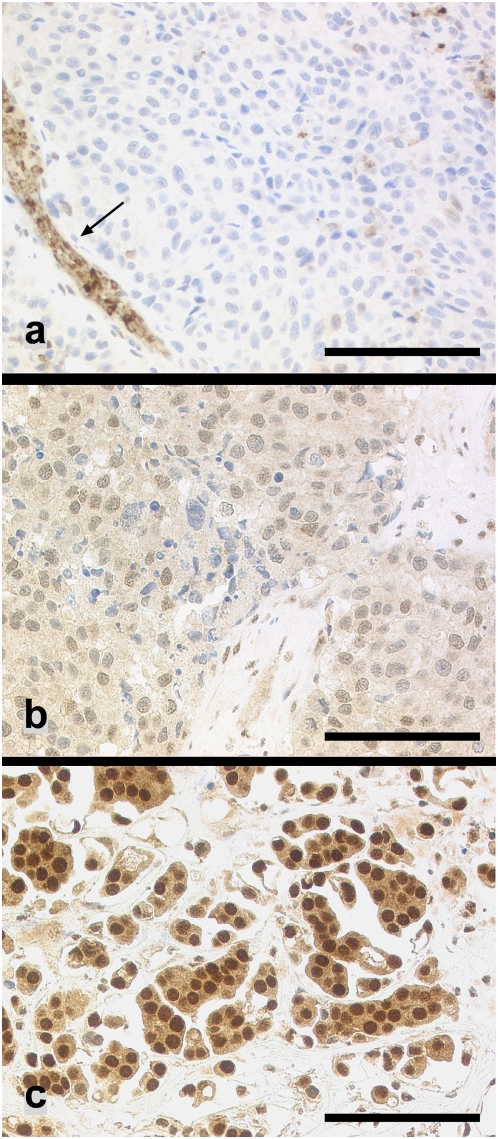
(a) Brain metastasis of a melanoma lacking MGMT immunoreactivity. MGMT-immunopositive endothelial cells and leukocytes served as internal positive control (arrow). (b) Heterogeneous MGMT immunoreactivity revealing MGMT-positive tumor cells intermingled with MGMT negative tumor cells (breast carcinoma). (c) Strong nuclear reaction for MGMT in all tumor cells (lung carcinoma). Scale bar: a–c: 100 µm.

MGMT-immunoreactivity was assessed of 285 brain metastases using a tissue microarray (77 lung carcinomas, 62 breast carcinomas, 42 renal cell carcinomas, 104 malignant melanomas). 96 of 285 (33.7%) tumor samples revealed a homogeneous MGMT expression (>95% MGMT-immunopositive tumor cells), whereas 91 of 285 (31.8%) lacked immunoreactivity for MGMT. In 98 cases (34.4%) a heterogeneous tumor population (1+ and 2+) was detectable consisting of MGMT-immunopositive and negative tumor cells. Examples are shown in **figure 2a**–**c**. The MGMT immunoreactivity pattern in the different tumor subgroups is shown in **table 1**. The fractions of 0 and 3+ samples varied significantly between the 4 tumor subtypes (**table 2**). 3+ samples were most frequent in breast and lung carcinoma metastases (46.8% and 42.6% respectively) whereas more than 45% of renal cell carcinoma and melanoma brain metastases were MGMT negative.

**Table 1:** MGMT immunoreactivity scores in the 4 tumor subgroups.

Tumor entity	Cases (n)	MGMT immunoreactivity n (%)			
		3+	2+	1+	0
**Renal cell carcinoma**	42	11 (26)	7 (17)	4 (10)	20 (47)
**Breast carcinoma**	62	29 (47)	17 (27)	12 (19)	4 (7)
**Lung carcinoma**	77	33 (43)	11 (14)	13 (17)	20 (26)
**Malignant melanoma**	104	23 (22)	15 (15)	19 (18)	47 (45)

**Table 2:** Differences between fractions of tumors with homogeneous (3+) and negative MGMT immunoreactivity in the various subgroups of brain metastases.

	Breast carcinoma	Lung carcinoma	Melanoma
**Renal cell carcinoma**	p<0.0001	p<0.025	n.s.
**Breast carcinoma**		p<0.025	p<0.0001
**Lung carcinoma**			p<0.01

### MGMT expression and MGMT promoter methylation status

**Figure 3:**
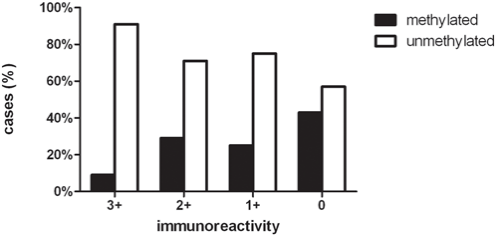
MGMT immunoreactivity and promoter methylation status in brain metastases. The fractions of brain metastases with unmethylated and methylated MGMT promoter differ significantly in 3+ versus 0 (p<0.001) and in 3+ versus 2+ (p = 0.0174) tumors.

Methylation status of the MGMT promoter as assessed by MS-PCR as well as the MGMT immunoprofile was available from 178 tumor samples (27 metastases of renal cell carcinoma, 39 of breast carcinoma, 36 of lung carcinoma, and 76 of malignant melanoma). There was a significant correlation between homogeneous MGMT-positivity and an unmethylated MGMT promoter. Forty-nine of 54 (90.7%) brain metastases displaying a homogeneous MGMT immunoreactivity revealed an unmethylated MGMT promoter. 21 of 28 (75%) brain metastases with a 1+ score and 25 of 35 (71.4%) brain metastases with a 2+ score had an unmethylated MGMT promoter. However, only 26 of 61 brain metastases (42.6%) lacking MGMT-immunoreactivity showed a methylated MGMT promoter. MGMT methylation frequencies in MGMT 3+, 2+, 1+ and 0 brain metastases are shown in **figure 3**.

A separate analysis of the individual tumor subgroups generally confirmed that MGMT immunopositivity correlates with an unmethylated MGMT promoter. The frequencies of tumors with a homogeneous MGMT staining and an unmethylated promoter ranged between 100% (7 of 7 clear cell renal cell carcinomas) and 86% (12 of 14 lung carcinomas). In contrast MGMT promoter methylation and lack of MGMT reactivity ranged between 67% (2 of 3 breast cancer) and 32% (11 of 34 melanomas). The detailed frequencies of MGMT reactivity and promoter methylation status in all tumor subgroups is shown in **table 3**.

**Table 3:** MGMT immunoreactivity and promoter methylation status in the individual tumor subgroups of brain metastases.

Tumor entity	MGMT promoter methylation status	Cases (n)	MGMT immunoreactivity, n (%)			
			3+	2+	1+	0
**Renal cell carcinoma**	methylated	6	0	0	0	6 (100)
	unmethylated	21	7 (33)	6 (29)	1(5)	7 (33)
**Breast carcinoma**	methylated	10	2 (20)	5 (50)	1 (10)	2 (20)
	unmethylated	29	16 (55)	8 (28)	4 (14)	1 (3)
**Lung carcinoma**	methylated	14	2 (14)	3 (22)	2 (14)	7 (50)
	unmethylated	22	12 (55)	2 (9)	4 (18)	4 (18)
**Malignant melanoma**	methylated	18	1(6)	2 (11)	4 (23)	11 (60)
	unmethylated	58	14 (24)	9 (16)	12 (21)	23 (39)

MGMT immunoreactivity and promoter methylation status were significantly associated in renal cell carcinoma (p<0.05), breast carcinoma (p<0.05) and lung carcinoma (p<0.025). A similar trend was seen in melanoma (p>0.05).

## Discussion

In this study, we demonstrate that about 30% of brain metastases originating from renal, breast and lung cancer as well as from malignant melanomas reveal a methylated MGMT-promoter. We found a strong correlation between a homogeneous MGMT expression pattern and an unmethylated MGMT promoter. In contrast MGMT negative brain metastases only in 42.6% showed a methylated MGMT promoter.

The therapeutic strategy to treat brain metastases depends on the patients' performance status, systemic tumor activity and the negative impact of older age. Treatment with surgery, radiosurgery and whole brain radiation therapy (WBRT) are the first line therapies for the majority of patients [11]. Although chemotherapy as a single modality has demonstrated limited efficacy, it may improve the result as a concurrent treatment [11]. Overall, there is only limited data on chemotherapeutic protocols from which no firm treatment recommendation can be drawn. Treatment efficacy is determined by the sensitivity of tumor cells to chemotherapeutic agents. Therefore, the chemotherapeutic regimen with highest efficacy to fight the primary tumor in principle is considered also to be the most efficacious for the corresponding brain metastasis [24]. In general, malignant melanoma, renal cell carcinoma and NSCLC show a fairly low chemosensitivity, whereas breast cancer reveal a moderately, SCLC and germ cell cancers a rather high chemosensitivity [11].

The role of TMZ in the treatment of brain metastases is still unclear. Several studies on treating brain metastases with TMZ alone showed low response rates. Preliminary results from randomized trials suggest that combination of TMZ and WBRT is an effective option for patients with brain metastases of non small cell lung cancer [25]. In malignant melanoma, a reduction of mortality from 69% to 41% was observed [26]. For patients with breast cancer [11] and renal cell carcinoma brain metastases [27], TMZ seems to be less helpful. An obvious possible explanation for variable TMZ efficacy in treating brain metastases is that MGMT promoter methylation has not been investigated systematically in brain metastases [28]. Thus, similarly as for malignant gliomas, where epigenetic silencing of the *MGMT* gene by promoter methylation has been shown to be of predictive value for profiting from TMZ [4], [5], [7], TMZ efficacy needs to be correlated to the MGMT promoter methylation status in individual brain metastases.

In this study, we show that about one third of brain metastases revealed a methylated MGMT promoter (29.6%; 59 of 199). The methylation rate in the different tumor subgroups (lung, breast, renal carcinoma and malignant melanoma) ranged between 20% and 46.5%. These results are in line with a previous study on a rather limited number of brain metastases (n = 28) resulting in promoter methylation in ∼36% [29].

Most studies assessing MGMT promoter methylation status utilize MS-PCR, which is a cost-efficient method requiring only small quantities of DNA. However, DNA derived from FFPE-tissue – the routine approach to process tissue for histological assessment and archiving - has been reported to be more often degraded, thus limiting the validity of molecular analyses. On top, bisulfite treatment – a prerequisite for MGMT promoter methylation assays - introduces various additional DNA strand breaks resulting in highly fragmented single stranded DNA [30]. Detection of the MGMT methylation status by 80 cycles of a nested PCR, as recommended for DNA isolated from formalin-fixed paraffin-embedded tissue [13], may easily increase the frequency of sampling error, thus negatively influencing the reliability of results obtained by MS-PCR. This may explain as to why only 61.2% (199 of 325) of our samples were evaluable by MS-PCR and why only in 75% of the cases replicate experiments on 20 randomly selected tumor samples yielded reproducible results. Despite such limitations MS-PCR on FFPE has been shown to be a valid and trustable technique resulting in reproducible data, which closely mirrors results obtained by MS-PCR on fresh frozen tissue [31].

High levels of endogenous MGMT in tumor cells are believed to protect the tumor from alkylating agents used in chemotherapeutic regimen and MGMT levels may be an important parameter of treatment failure. Therefore, some investigators prefer the somewhat simpler immunohistochemical approach to detect the expression of the MGMT protein [14]–[16]. Compared to MS-PCR, immunohistochemistry is a more reliable method if only FFPE tissue is available. However, the relevance of MGMT-immunoreactivity is a matter of intense discussion especially when MGMT-immunoreactivity is correlated to MGMT promoter methylation status [17]–[19], [32], [33]. We therefore evaluated 285 brain metastases for MGMT expression by immunohistochemistry. In about one third of the cases more than 95% of the tumor cells were MGMT-immunopositive, whereas in one third no immunoreactivity was detectable. The remaining cases showed a heterogeneous MGMT-immunoprofile ranging from 5 to 95%. In 178 cases, MS-PCR and immunohistochemical data was available. We found a strong correlation between homogeneous MGMT-immunoreactivity and unmethylated MGMT promoter. MGMT-immunoreactivity and evidence of promoter methylation in 9% of the samples may reflect differences in the methylation status of the MGMT promoter in tumor cell subpopulations as it is reported for malignant melanoma [34].

Furthermore, extensive MGMT promoter methylation has been shown to go along with *MGMT* gene expression under certain conditions [35]. A negative MGMT-immunostaining, however, was not correlated with a defined promoter methylation status, possibly because methylation of the MGMT promoter is not necessarily linked to MGMT protein expression. Other mechanisms of gene silencing including gene deletion or mutation may lead to loss of protein expression - with or without promoter methylation. Moreover, MGMT is an inducible protein [33], [36], [37] and lack of immunoreactivity at time of diagnosis might not reflect the potential functionality of the protein.

MS-PCR proposes a clear MGMT promoter methylation status and divides the tumor samples into PCR-positive and –negative cases. However, the regulation of MGMT expression is a more complex phenomenon in which methylation of the promoter is not the only determining factor [38], [39]. For instance, in in vitro experiments wild-type p53 seems to act as an inhibitor of MGMT expression, suggesting tumors with normal p53 would have more likely low or absent MGMT levels, independent of promoter methylation. On the other hand it has been suggested that mutant p53 may be associated with a decreased MGMT expression and/or methylation [40], [41]. Given the different relevance of p53 alterations in melanoma or breast, lung and renal cancer, such mechanisms may explain the tumor type-specific differences of MGMT immunoreactivity between these tumors **(** **table 2** **)**. Assessing the protein, e.g. by immunohistochemistry, bypasses several of the above-mentioned pitfalls.

There are at least a few studies on malignant gliomas which corroborate that MGMT-immunoreactivity is associated with survival and/or response to alkylating substances [14]–[16], [42], [43]. For example, patients with high MGMT expression were reported to have a lower response rate when receiving TMZ before radiotherapy. Based on such reports one may hypothesize that MGMT-immunoreactivity may be a negative predictor of treatment success with alkylating substances. However, the extent to which MGMT influences the treatment of brain metastases with alkylating agents needs to be explored in future studies.

In conclusion, we demonstrate that about one third of brain metastases of various origins revealed a methylated MGMT promoter as assessed by MS-PCR assay. This suggests that brain metastases may be a potential target for therapy with alkylating substances. Showing a clear correlation between homogeneous MGMT immunoreactivity and an unmethylated MGMT promoter, we hypothesize that MGMT immunohistochemistry – as a screening method - could be a helpful diagnostic tool to identify those tumors that probably will not benefit from the use of alkylating agents like temozolomide. Clinical data is necessary to validate this hypothesis. However, the discrepancy between promoter methylation and MGMT negativity necessitates combined immunostaining and methylation specific PCR.
